# Inhibition of protein kinase C enhances angiogenesis induced by platelet-derived growth factor C in hyperglycemic endothelial cells

**DOI:** 10.1186/s12933-015-0180-9

**Published:** 2015-02-07

**Authors:** Junji Moriya, Napoleone Ferrara

**Affiliations:** Moores Cancer Center, University of California, 3855 Health Sciences Dr, La Jolla, San Diego, CA 92093 USA

**Keywords:** Platelet-derived growth factor C (PDGF-C), Diabetes, Therapeutic angiogenesis, Protein kinase C (PKC), Hyperglycemia, Endothelial cells

## Abstract

**Background:**

Diabetes is a risk factor for the development of cardiovascular diseases with impaired angiogenesis. We have previously shown that platelet-derived growth factor C (PDGF-C) and its receptor, PDGF receptor α (PDGFR-α) were downregulated in ischemic limbs of diabetic mice, although the underlying mechanisms remained elusive. Protein kinase C (PKC) is a family of serine/threonine kinases and is known to be involved in angiogenesis. The purpose of this study is to elucidate the mechanisms of how PDGF-C/PDGFR-α axis is impaired in diabetes.

**Methods:**

Human umbilical vein endothelial cells (HUVECs) and human cardiac microvascular endothelial cells (HMVECs) cultured in normoglycemic or hyperglycemic conditions were examined. We also examined the effects of PKC inhibition on the PDGF-C/PDGFR-α axis in endothelial cells exposed to hyperglycemia.

**Results:**

Hyperglycemia inhibited proliferation and decreased viability of both HUVECs and HMVECs. Hyperglycemic endothelial cells exhibited decreased PDGFR-α expression both at messenger RNA (mRNA) and protein levels, while there was no significant change in expression of PDGF-C. We also found that expression of PKC-α, one of the PKC isoforms, was increased in hyperglycemic endothelial cells and that inhibition of PKC upregulated PDGFR-α expression in these cells. Phosphorylation of extracellular signal-regulated kinase (ERK) and Akt induced by PDGF-C was significantly attenuated in hyperglycemic endothelial cells, whereas inhibition of PKC effectively reversed these inhibitory effects. Moreover, inhibition of PKC also promoted angiogenesis induced by PDGF-C in hyperglycemic endothelial cells, which was not observed in vascular endothelial growth factor-A (VEGF-A)-induced angiogenesis.

**Conclusions:**

These findings suggest that downregulation of the PDGF-C/PDGFR-α axis is involved in impaired angiogenesis of hyperglycemia through upregulation of PKC. Targeting PKC to restore PDGF-C signaling might be a novel therapeutic strategy for the treatment of vascular complications in diabetes.

**Electronic supplementary material:**

The online version of this article (doi:10.1186/s12933-015-0180-9) contains supplementary material, which is available to authorized users.

## Introduction

There are increasing numbers of patients who suffer from ischemic cardiovascular diseases, and most of such patients have some risk factors. Among them, diabetes is known to have strong association with the development of cardiovascular diseases [1,2]. Indeed, vascular complications associated with diabetes may result from atherosclerosis of large vessels, causing cardiac, cerebral and peripheral vascular diseases [3,4]. Moreover, these macrovascular atherosclerotic diseases are often refractory to conventional therapies, leading to increased morbidity and mortality among patients of diabetes [5-7]. Thus, it is important to establish novel therapeutic modalities for these patients.

Therapeutic angiogenesis is a relatively new and promising concept for treating patients with ischemic cardiovascular diseases [8-10]. It involves the use of angiogenic growth factors to promote development of collateral arteries. Vascular endothelial growth factor-A (VEGF-A) has been used as a major tool of this therapy since it is considered as the key regulator of angiogenesis [11-14]. However, several lines of evidence have shown that the benefits of this treatment are limited [15]. Indeed, the angiogenic response after ischemia is attenuated in patients with diabetes, interfering with the response to treatment [16,17]. Moreover, it has recently been reported that a ligand-independent VEGF receptor 2 (VEGFR2) signaling pathway is activated in diabetic endothelial cells, leading to impaired responses to exogenous VEGF-A and limited angiogenic events [18,19]. Collectively, these studies suggest that further basic research is needed to elucidate the mechanisms of angiogenesis, especially in diabetic state, to improve the overall outcome of therapeutic angiogenesis.

Platelet-derived growth factors (PDGFs) are potent mitogenic and migratory factors for many cell types of mesenchymal origin [20]. The PDGF family consists of four different polypeptide chains encoded by four different genes: PDGF-A, PDGF-B, and the more recently discovered PDGF-C and PDGF-D [21,22]. Among these, PDGF-C has been known to promote angiogenesis independently of VEGF-A [23]. Actually, inhibition of PDGF-C leads to reduced angiogenesis in experimental tumors refractory to anti-VEGF treatment [24] or to suppression of both choroidal and retinal neovascularization [25]. Moreover, PDGF-C has been reported to revascularize ischemic tissues by mobilizing endothelial progenitor cells or stimulating migration of endothelial cells [26]. We also have recently found that expression of PDGF-C and its receptor, PDGF receptor α (PDGFR- α) was downregulated in ischemic limb tissues of diabetic mice, possibly contributing to impaired angiogenesis of diabetes. Moreover, introduction of PDGF-C significantly promoted revascularization in these mice [27]. Collectively, these studies suggest that PDGF-C may be an interesting candidate for therapeutic angiogenesis. However, the underlying mechanisms of how PDGF-C/PDGFR-α axis is impaired in diabetes still remain to be elucidated.

Therefore, we sought to investigate such mechanisms by using human endothelial cells (ECs) exposed to hyperglycemic condition. Hyperglycemia inhibited cell proliferation and decreased cell viability, which is in agreement with a previous study [28]. Moreover, we found that the expression of PDGFR-α was downregulated in hyperglycemic ECs both at mRNA and protein levels. We also found that upregulation of protein kinase C (PKC)-α expression was involved in decreased PDGFR-α expression in hyperglycemic ECs. Consistent with this finding, inhibition of PKC led to augmentation of intracellular signaling induced by PDGF-C, resulting in promotion of angiogenesis in hyperglycemic ECs. These findings suggest that downregulation of the PDGF-C/PDGFR-α axis is involved in impaired angiogenesis of diabetes through upregulation of PKC. Targeting PDGF-C and PKC might be a novel strategy for therapeutic angiogenesis in the diabetic state.

## Materials and methods

### Reagents

Recombinant human VEGF-A and PDGF-C were from Peprotech (Rocky Hill, NJ) and Sigma-Aldrich (St. Louis, MO), respectively. D-mannitol and d-glucose were from Sigma-Aldrich. Trypan blue solution was from GE Healthcare Life Sciences (Little Chalfont, UK). Bisindolylmaleimide I (Bis I) was purchased from Cell Signaling Technology (Danvers, MA). Sulforaphane was from Trevigen (Gaithersburg, MD).

### Cells

Human umbilical vein endothelial cells (HUVECs), human cardiac microvascular endothelial cells (HMVECs), and human aortic smooth muscle cells (AoSMCs) were purchased from Lonza (Walkersville, MD). HUVECs were grown in EGM-2 medium (Lonza), HMVECs in EGM-2-MV medium (Lonza), and AoSMCs in SmGM-2 medium (Lonza) on gelatin-coated tissue culture dishes (BD Bioscience, Becton, NJ), respectively. Cells from fewer than 5 generations were used for all experiments. High-glucose cultures were grown in the presence of 30 mM glucose for a minimum of 5 days and then subjected to further analysis. The total cell numbers were calculated by a Countess automated cell counter (Life Technologies, Carlsbad, CA). For signaling pathway analysis and tube formation analysis, HUVECs were serum-starved for 15 hours in EBM-2 medium (Lonza) supplemented with 0.1% fetal bovine serum (Omega Scientific, Tarzana, CA) before being treated with test substances.

### RNA analysis

Total RNA was extracted by an RNeasy Plus Mini kit (Qiagen, Valencia, CA) according to the manufacturer’s instructions. cDNA was prepared using High Capacity cDNA Reverese Transcription Kits (Life Technologies). Quantitative real-time polymerase chain reaction (PCR) was performed by the ViiA7 Real-Time PCR system (Life Technologies) with the Taqman Gene Expression Assays and the Taqman Fast Advanced Master Mix (Life Technologies). Glyceraldehyde-3-phosphate dehydrogenase (GAPDH) messenger RNA was used as an endogenous control for quantitative real-time PCR analyses. At least three biological replicates were included in each analysis.

### Western blot analysis

Whole cell lysates were prepared in RIPA buffer (Thermo Scientific, Rockford, IL) with Halt Protease and Phosphatase Inhibitor Cocktail (Thermo Scientific). The lysates (20 to 40 μg) were resolved by SDS-PAGE. The proteins were then transferred into a polyvinylidene difluoride membrane (Bio-Rad, Hercules, CA) and incubated with the primary antibody followed by anti-rabbit IgG-horseradish peroxidase antibody or anti-mouse IgG-horseradish peroxidase antibody (GE Healthcare, Little Chalfont, UK). Specific proteins were detected by the enhanced chemiluminescent substrate (Thermo Scientific). The primary antibodies used for Western blotting were as follows: anti-PDGFRα antibody (951, sc-431), anti-PKCα antibody (C-20, sc-208), anti-PKCβΙΙ antibody (C-18, sc-210), anti-PKCδ antibody (C-17, sc-213) (Santa Cruz Biotechnology, Santa Cruz, CA), anti-phospho-extracellular signal-regulated kinase (ERK) antibody (20G11, #4376), anti-ERK antibody (137F5, #4695), anti-phospho-Akt antibody (D9E, #4060), anti-Akt antibody (C67E7, #4691) (Cell Signaling Technology), and anti-β-actin antibody (AC-74, A2228) (Sigma-Aldrich).

### Tube formation assay

The tube formation assay was performed using a commercially available kit (*In Vitro* Angiogenesis Tube Formation Assay Kit, Trevigen, #3470-096-K) according to the manufacturer’s instructions. Briefly, growth factor-reduced basement membrane extract solution in a 96-well plate was allowed to form a reconstituted matrix for one hour at 37°C. HUVECs were seeded at 1.5 × 10^4^ per well and cultured for up to 24 hours in the presence or absence of different kinds of test substances. Capillary-like tube formation was assessed by photography under a stereoscopic microscope (Zeiss, Oberkochen, Germany) at a ×80 magnification. Total tube length was analyzed by using Image J software (NIH, Bethesda, MD).

### Statistical analysis

The data are shown as means ± standard error of the mean. Differences between two groups were analyzed by a two-sided Student *t*-test. One-way analysis of variance (ANOVA) was used for multiple group comparison followed by the Bonferroni procedure for comparison of means. All experiments were repeated at least three times. In all analyses, *P* values of < 0.05 were considered statistically significant.

## Results

### Hyperglycemia inhibits cell proliferation and decreases cell viability of endothelial cells

**Figure 1:**
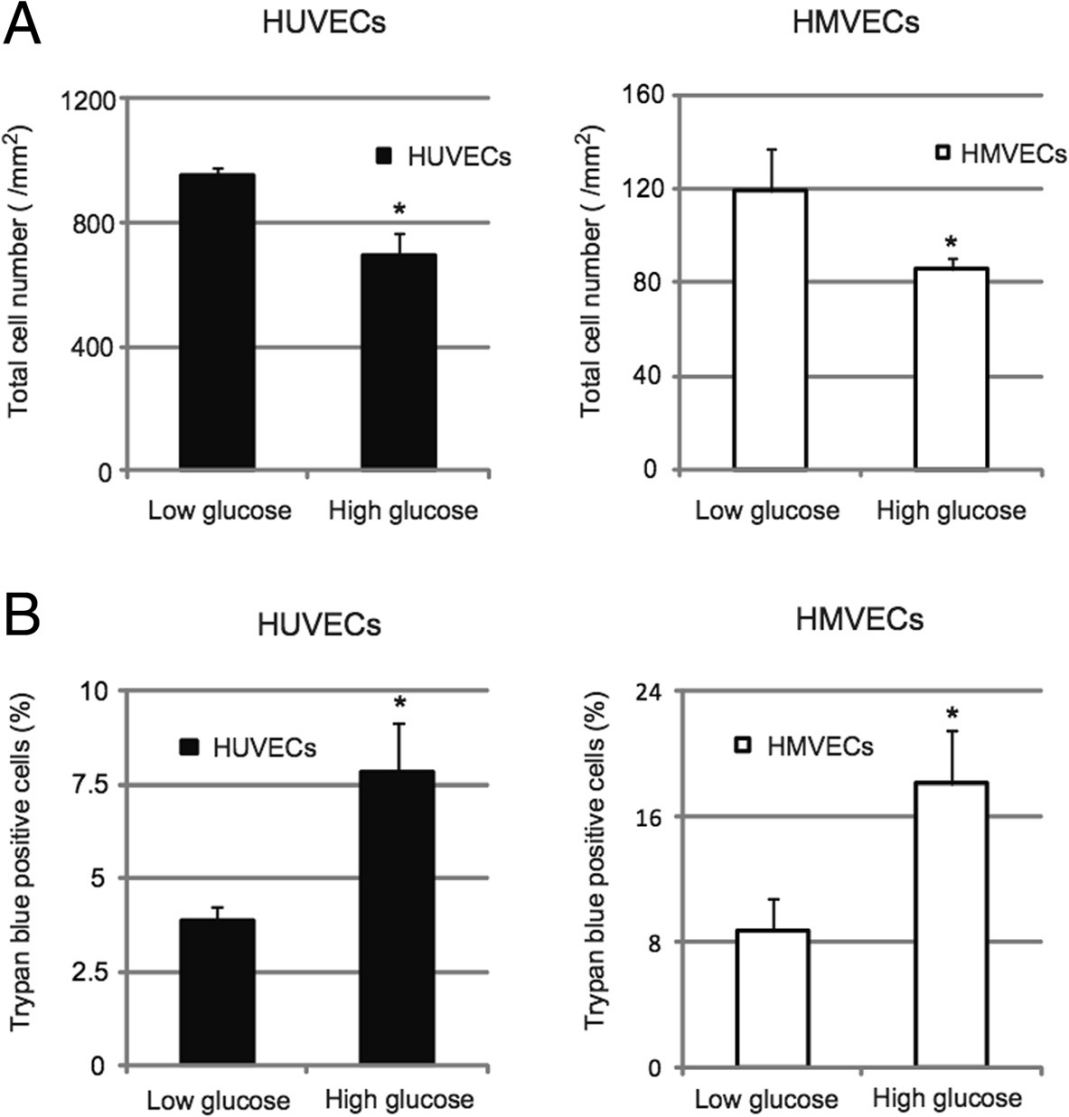
**Effects of hyperglycemia on endothelial cells. A**: HUVECs or HMVECs were treated with 5.5 mM (Low) or 30 mM (High) glucose and total cell numbers were calculated. *P < 0.05 vs Low glucose (n = 4 for each group). Data represent means ± standard error of the mean. **B**: Ratio of trypan blue positive HUVECs or HMVECs treated with 5.5 mM (Low) or 30 mM (High) glucose. *P < 0.05 vs Low glucose (n = 4 for each group). Data represent means ± standard error of the mean.

We first examined the effect of hyperglycemia on proliferation and viability of endothelial cells (ECs). Since we confirmed that the total number of cells and the ratio of cells positive for trypan blue staining exposed to 24.5 mM d-mannitol in normoglycemic (5.5 mM d-glucose) conditions were not different from those in control cultures (Additional file 1: Figure S1), we used 24.5 mM d-mannitol as an osmotic control for in all further experiments. We examined two types of human EC; human umbilical vein endothelial cells (HUVECs) and human cardiac microvascular endothelial cells (HMVECs). We plated HUVECs and HMVECs in normoglycemic or hyperglycemic (30 mM d-glucose) conditions and cultured them for 5 days. As shown in Figure 1A, HUVECs or HMVECs cultured for 5 days in such hyperglycemic condition showed reduced increases in total cell numbers, compared to normoglycemic conditions. Moreover, the ratio of cells positive for trypan blue staining, which are thought to be dead cells, was significantly increased in HUVECs and HMVECs cultured in hyperglycemic condition (Figure 1B). These results suggest that hyperglycemia inhibits cell proliferation and decreases cell viability of ECs.

### Expression of PDGFR-α is downregulated in hyperglycemic endothelial cells

**Figure 2:**
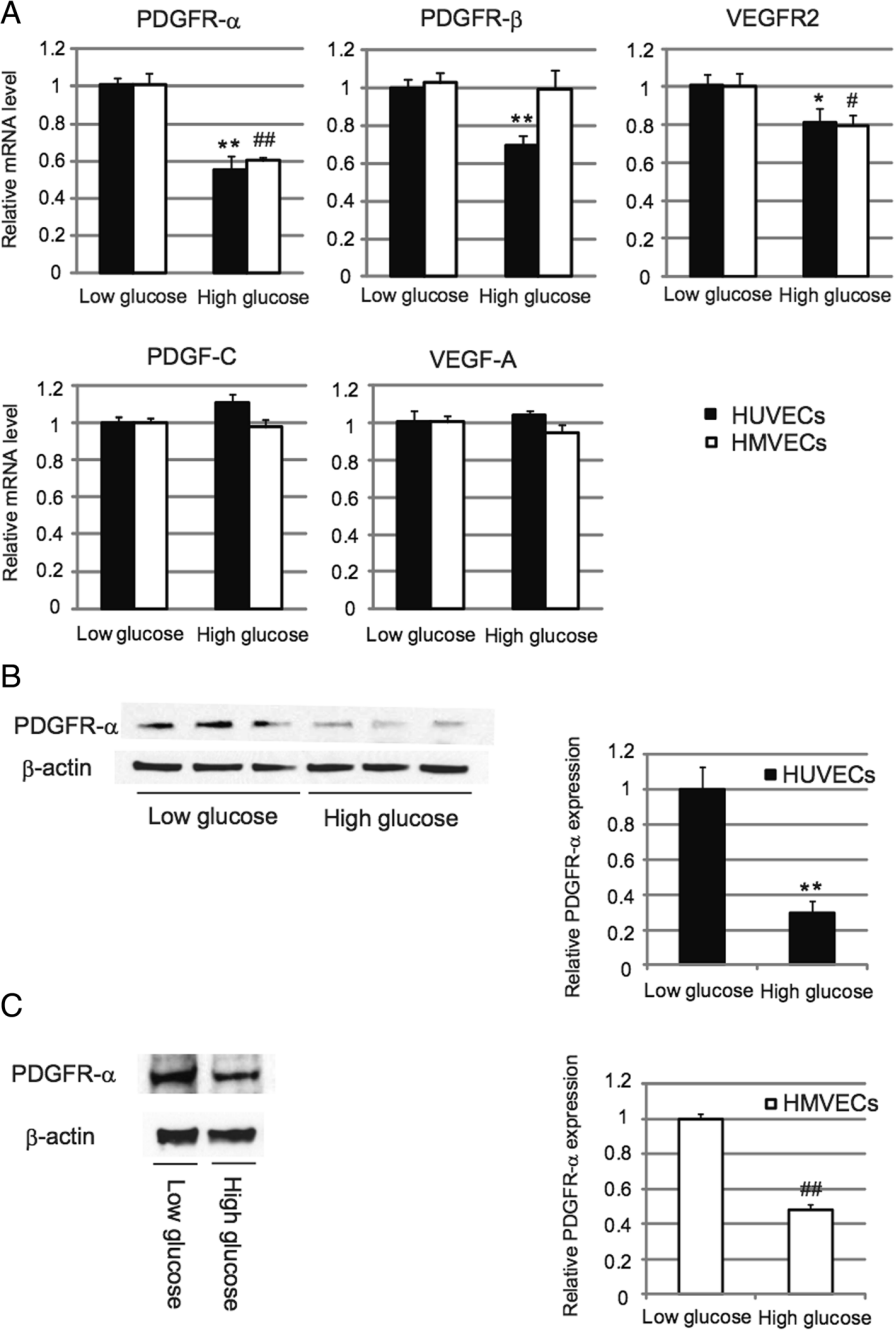
**Expression of PDGFR-α is downregulated in hyperglycemic endothelial cells. A**: PDGFR-α, PDGFR-β, VEGFR2, PDGF-C, and VEGF-A mRNA levels in HUVECs or HMVECs treated with 5.5 mM (Low) or 30 mM (High) glucose were assessed by quantitative real-time PCR. *P < 0.05, **P < 0.01 vs Low glucose HUVECs (n = 6 for each group). #*P* < 0.05, ##*P* < 0.01 vs Low glucose HMVECs (n = 6 for each group). Data represent means ± standard error of the mean. **B**: Western blot analysis of PDGFR-α expression in HUVECs treated with 5.5 mM (Low) or 30 mM (High) glucose. Relative quantification data are also shown (right). **P < 0.01 vs Low glucose (n = 6 for each group). Data represent means ± standard error of the mean. **C**: Western blot analysis of PDGFR-α expression in HMVECs treated with 5.5 mM (Low) or 30 mM (High) glucose. Relative quantification data are also shown (right). ##*P* < 0.01 vs Low glucose (n = 8 for each group). Data represent means ± standard error of the mean.

We have previously reported that expression of PDGF-C or PDGFR-α after ischemia is decreased in diabetic mice, leading to impaired angiogenesis [27]. Thus, we sought to examine messenger RNA (mRNA) levels of these angiogenic factors in hyperglycemic ECs (HUVECs and HMVECs) by quantitative real-time PCR (qRT-PCR) analysis. We found that compared to normoglycemic conditions, expression of PDGFR-α was markedly decreased in hyperglycemic ECs (Figure 2A). VEGFR2 expression was also modestly decreased in hyperglycemic ECs, although we did not observe any differences in PDGF-C or VEGF-A expression compared to normoglycemia (Figure 2A). After confirming that PDGFR-α expression was almost comparable in normoglycemic ECs treated with or without 24.5 mM d-mannitol (Additional file 2: Figure S2A, B), we performed Western blot analysis to examine the protein expression of PDGFR-α in hyperglycemic ECs. Consistent with our qRT-PCR results, the expression of PDGFR-α in hyperglycemic ECs was markedly decreased compared to normoglycemic, mannitol-treated ECs (Figure 2B, C). These results indicate that the expression of PDGFR-α is downregulated in hyperglycemic ECs both in mRNA and protein levels.

### Upregulation of PKC leads to decreased PDGFR-α expression in hyperglycemic endothelial cells

**Figure 3:**
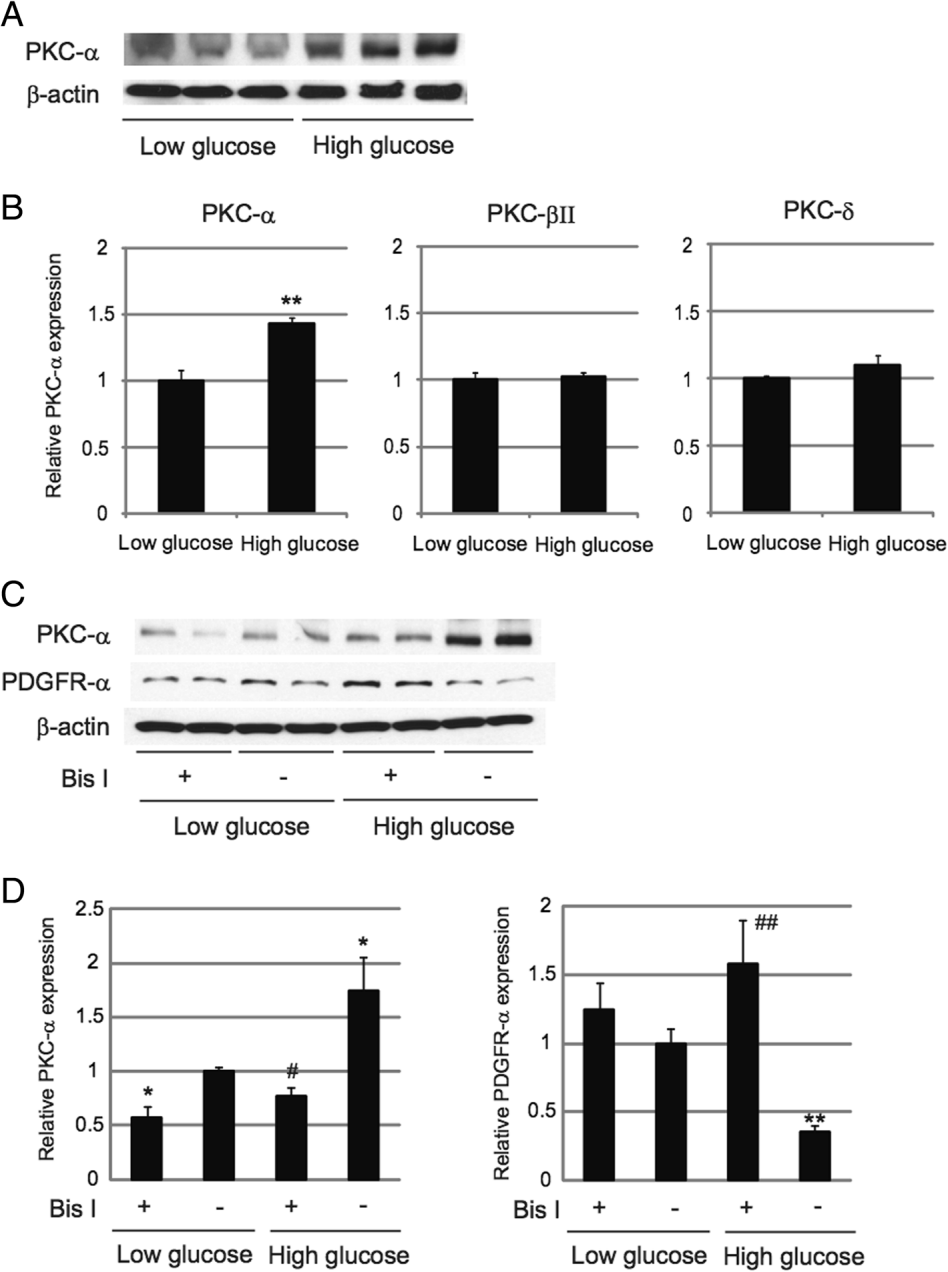
**Upregulation of PKC leads to decreased PDGFR-α expression in hyperglycemic endothelial cells. A**: Western blot analysis of PKC-α in HUVECs treated with 5.5 mM (Low) or 30 mM (High) glucose. **B**: Relative quantification data of Western blot analysis of PKC-α, PKC-βII, and PKC-δ in HUVECs treated with 5.5 mM (Low) or 30 mM (High) glucose. **P < 0.01 vs Low glucose (n = 4 for each group). Data represent means ± standard error of the mean. **C**: HUVECs exposed to 5.5 mM (Low) or 30 mM (High) glucose were treated with or without bisindolylmaleimide I (Bis I) at a concentration of 4 μΜ for 30 minutes and then were subject to Western blot analysis for PKC-α and PDGFR-α expression. **D**: Relative expression levels of PKC-α and PDGFR-α in the experiment of Figure 3C. *P < 0.05, **P < 0.01 vs Low glucose, Bis I (−) group. #*P* < 0.05, ##*P* < 0.01 vs High glucose, Bis I (−) group. n = 4 for each group. Data represent means ± standard error of the mean.

Hyperglycemia has been reported to activate members of the protein kinase C (PKC) family, some of which are implicated in angiogenesis [29]. We hypothesized that PKC might be implicated in the decreased expression of PDGFR-α in hyperglycemic ECs. To test this hypothesis, we first examined the expression of PKC-α, PKC-βΙΙ, and PKC-δ, which are known to be expressed in ECs [30]. Because HUVECs and HMVECs showed the same tendency toward the total number of cells, ratio of cells positive for trypan blue staining, and expression of PDGFR-α exposed to hyperglycemic conditions (Figures 1, 2), and considering that HUVECs have been widely used to elucidate molecular mechanisms of diabetic state in ECs [31,32], we used only HUVECs for further experiments. We found that hyperglycemia significantly upregulated the protein expression of PKC-α in HUVECs (Figure 3A, B). On the other hand, the expression of PKC-βΙΙ or PKC-δ was not significantly different between normoglycemic and hyperglycemic ECs (Figure 3B), although we could not exclude the possibility that these isoforms were still activated in hyperglycemia. Next, we investigated the effects of inhibition of PKC. We treated normoglycemic and hyperglycemic HUVECs with bisindolylmaleimide I (Bis I), an inhibitor of PKC [33], and examined the expression of PKC-α and PDGFR-α by Western blot analysis. Consistent with previous obsevations [34], we confirmed that treatment with Bis I significantly downregulated the expression of PKC-α both in normoglycemic and hyperglycemic HUVECs (Figure 3C, D). Interestingly, downregulation of PDGFR-α expression was completely abolished by treatment with Bis I in hyperglycemic ECs (Figure 3C, D). We also confirmed that the effect of Bis I had some dose-dependent tendency to the reduced expression of PKC-α, although it was not statistically significant (Additional file 3: Figure S3A). Moreover, we found that longer exposure to Bis I significantly upregulated the expression of PDGFR-α even in normoglycemic ECs (Additional file 3: Figure S3B). These results suggest that upregulation of PKC expression induced by hyperglycemia is involved in the downregulation of PDGFR-α expression in HUVECs.

### Inhibition of PKC leads to increased intracellular signaling induced by PDGF-C in endothelial cells

**Figure 4:**
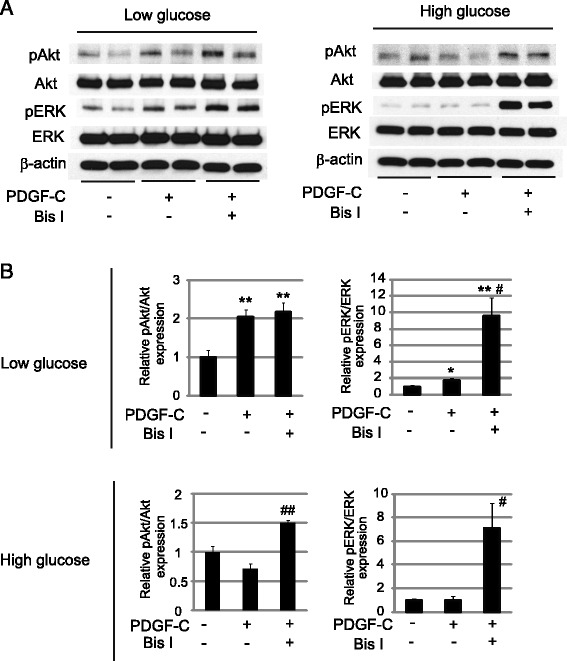
**Inhibition of PKC potentiates intracellular signaling induced by PDGF-C in endothelial cells. A**: HUVECs exposed to 5.5 mM (Low) or 30 mM (High) glucose were treated with PDGF-C (50 ng/mL) or PDGF-C + Bis I (4 μΜ) and analyzed for signaling molecules by Western blot analysis. **B**: Relative expression levels of pERK and pAkt in the experiment of Figure 4A. Results are shown as the ratio of pAkt to Akt densities, and pERK to ERK densities, respectively. *P < 0.05, **P < 0.01 vs PDGF-C (−), Bis I (−) group. #*P* < 0.05, ##*P* < 0.01 vs PDGF-C (+), Bis I (−) group. n = 4 for each group. Data represent means ± standard error of the mean.

Next we investigated whether downregulation of PDGFR-α in hyperglycemic ECs actually affects downstream signaling induced by PDGF-C. We found that PDGF-C-induced phosphorylation of Akt and ERK, both of which are crucial kinases in the intracellular signaling pathway for this growth factor, was abolished in hyperglycemic HUVECs, which is in contrast to normoglycemic HUVECs (Figure 4A, B). However, pretreatment with Bis I significantly increased phosphorylation of Akt in hyperglycemic HUVECs and phosphorylation of ERK in hyperglycemic and normoglycemic HUVECs following PDGF-C stimulation (Figure 4A, B). We also observed decreased VEGF-A-induced phosphorylation of Akt and ERK in hyperglycemic HUVECs (Additional file 4: Figure S4). However, unlike PDGF-C, treatment with Bis I in hyperglycemic HUVECs did not increase phosphorylation of Akt or ERK induced by VEGF-A (Additional file 4: Figure S4). Moreover, tretament with Bis I significantly reduced VEGF-A-induced phosphorylation of Akt and ERK in normoglycemic HUVECs (Additional file 4: Figure S4). Collectively, these results suggest that inhibition of PKC could potentiate the intracellular signaling induced by PDGF-C, but not VEGF-A, in hyperglycemic HUVECs.

### Inhibition of PKC promotes angiogenesis induced by PDGF-C, but not VEGF-A, in hyperglycemic endothelial cells

**Figure 5:**
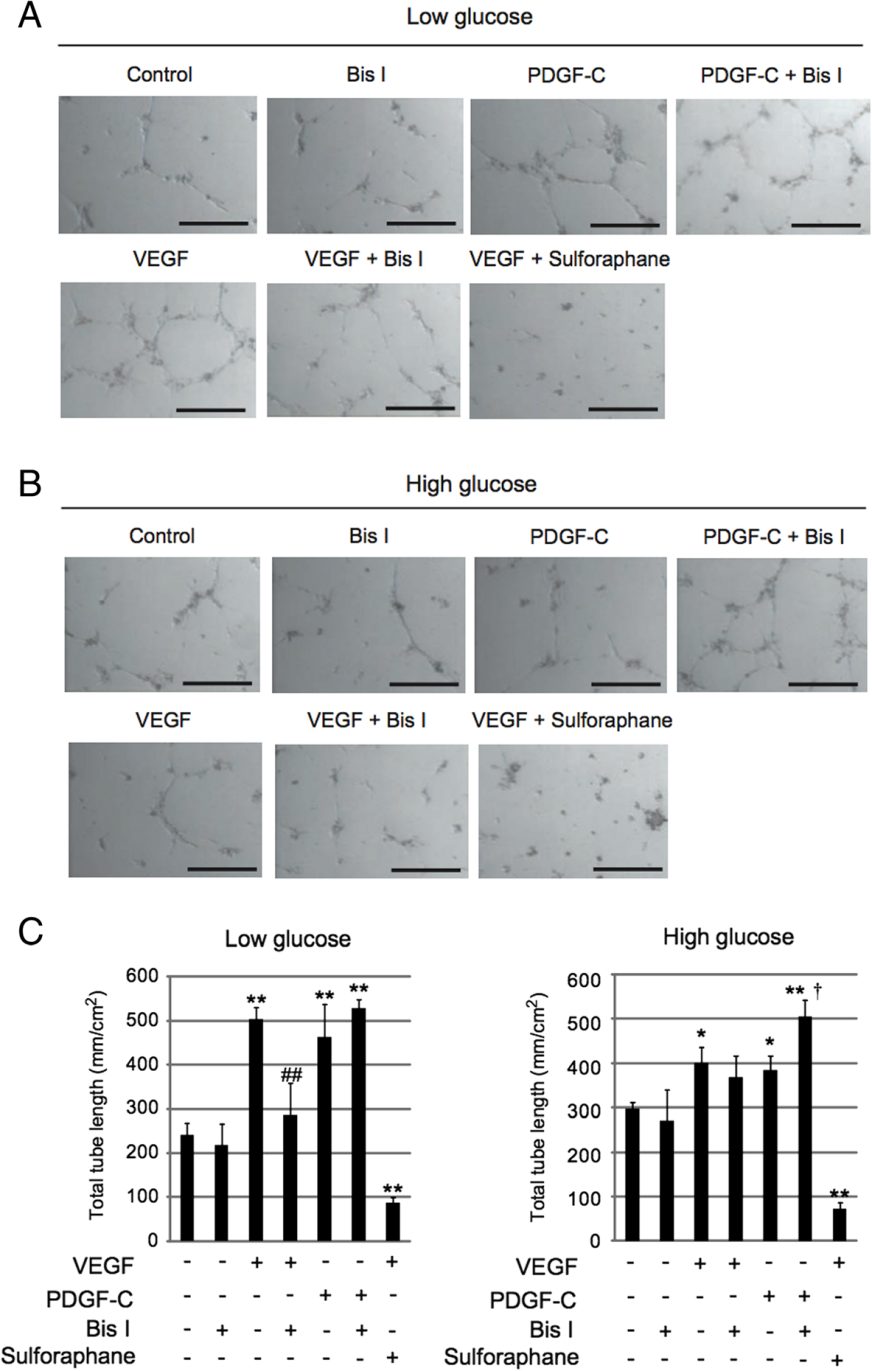
**Inhibition of PKC promotes angiogenesis induced by PDGF-C, but not VEGF-A, in hyperglycemic endothelial cells. A**, **B**: Photographs show tube formation in HUVECs exposed to 5.5 mM (Low) **(A)** or 30 mM (High) **(B)** glucose in the presence of Bis I (4 μΜ), PDGF-C (50 ng/mL), PDGF-C + Bis I, VEGF (50 ng/mL), VEGF + Bis I, and VEGF + Sulforaphane (5 μΜ). PBS treatment served as control (Control). Sulforaphane was used as a negative control for the assay. Scale bar = 300 μm. **C**: Total tube length was analyzed in the experiment of Figure 5A (left) and 5B (right). *P < 0.05, **P < 0.01 vs Control (VEGF(−), PDGF-C(−), Bis I(−), and Sulforaphane(−)) group. ##*P* < 0.01 vs VEGF(+), PDGF-C(−), Bis I (−), and Sulforaphane (−) group. †*P* < 0.05 vs VEGF(−), PDGF-C(+), Bis I (−), and Sulforaphane (−) group. n = 5 ~ 9 for each group. Data represent means ± standard error of the mean.

To investigate the effects of PKC inhibition on angiogenesis, we performed tube formation assays using HUVECs in normoglycemic and hyperglycemic conditions. Sulforaphane was used as a negative control for the assays. We found that both PDGF-C and VEGF-A treatment markedly increased tube formation in normoglycemic HUVECs, while this increase was blunted but still significant in hyperglycemic HUVECs (Figure 5A-C). Intriguingly, treatment with Bis I significantly augmented PDGF-C-induced tube formation in hyperglycemic HUVECs (Figure 5B, C). However, treatment with Bis I did not increase or even significantly decreased VEGF-A-induced tube formation (Figure 5A-C). Treatment with Bis I alone did not affect tube formation, both in normoglycemic and hypeglycemic HUVECs (Figure 5A-C). These results suggest that PKC inhibition promotes angiogenesis induced by PDGF-C, but not VEGF-A, in hyperglycemic HUVECs possibly by upregulating PDGF-C/PDGFR-α axis.

## Discussion

PDGFs have a variety of effects in many cell types. Among the four members of the PDGF family, PDGF-B and PDGF-C have been extensively characterized for their role in vessel maturation and growth. While PDGF-B requires other angiogenic factors like VEGF-A to induce neovascularization, PDGF-C has the potential to promote revascularization after ischemia, independently of other factors [23,26]. Moreover, we recently reported that expression of PDGF-C or PDGFR-α was downregulated in ischemic tissues of diabetic mice, resulting in the impaired blood flow recovery after ischemia [27]. Also, a recent study reported that PDGF signaling is critically involved in the pathogenesis of diabetes [35]. On the other hand, a clinical study revealed that there were no significant differences in the serum concentrations of VEGF between control subjects and diabetic patients [36]. Taken together, these findings support the hypothesis that PDGF-C might be a promising new target for the treatment of vascular complications related to diabetes.

The present study sought to investigate how the PDGF-C/PDGFR-α axis is downregulated using cultured human ECs exposed to hyperglycemia. We found that hyperglycemia led to inhibition of cell proliferation, decreased cell viability, and reduced angiogenic responses to VEGF-A in ECs (Figures 1A-B, 5A-C). These results are consistent with our previous *in vivo* studies in diabetic mice showing reduced blood flow recovery and neovascularization after ischemia even with introduction of VEGF-A [27,31].

We also show here that expression of PDGFR-α was significantly downregulated in hyperglycemic ECs, while expression of PDGF-C was almost unchanged between normoglycemic and hyperglycemic ECs (Figure 2A). On the other hand, we found that expression of PDGF-C was significantly downregulated both at baseline and after ischemia in limb tissues of diabetic mice in our previous *in vivo* study [27]. Since *in vivo* murine limb tissues contain various cell types besides ECs such as vascular smooth muscle cells, fibroblasts, and skeletal muscle cells, it is possible that decreased PDGF-C expression occurs in cell types other than ECs in diabetic limbs. Indeed, we observed that the expression of PDGF-C was decreased in hyperglycemic human aortic smooth muscle cells (AoSMCs) *in vitro* (data not shown), suggesting that SMCs might be more responsible for the decreased expression of PDGF-C *in vivo* as well. Further studies are required to identify the cellular localization of the decreased PDGF-C and/or PDGFR-α expression *in vivo*.

Our results also suggest that PKC is critically involved in the decreased PDGFR-α expression in hyperglycemic ECs. We found that expression of PKC-α was significantly upregulated in hyperglycemic ECs (Figure 3A, B). PKC is a member of a large family of serine/threonine kinases and is known to be involved in angiogenesis [37]. Moreover, PKC is also implicated in the increased risk of atherosclerosis in diabetes [38]. However, the role of PKC for angiogenesis still remains elusive because the effect of PKC on angiogenesis seems to be different or even opposite depending on PKC isoforms [39]. Moreover, there are some conflicting reports indicating that PKC-α promotes [40,41] or inhibits [42] angiogenic activity of ECs, and it has been known that inhibition of PKC leads to suppression of VEGF release from platelets [43]. We show here that inhibition of PKC by Bis I leads to increased PDGFR-α expression, resulting in potentiation of intracellular signaling, and augmentation of angiogenesis induced by PDGF-C, but not by VEGF-A (Figures 3, 4, 5). Based on these findings, we speculate that the complexity and apparent discrepancies in the effects of PKC on angiogenesis stem not only from different PKC isoforms but also from downstream molecular targets of PKC in ECs. Further studies are needed to confirm whether PKC differentially regulates angiogenesis induced by PDGF-C and VEGF-A signaling, in addition to the effects of specific inhibition or activation of PKC-α on angiogenesis induced by PDGF-C in the diabetic state.

In summary, the present study shows that downregulation of PDGF-C/PDGFR-α axis is involved in impaired endothelial cell functions in hyperglycemia at least in part through upregulation of PKC. Therefore, targeting PKC to restore PDGF-C signaling might be anovel therapeutic option for the treatment of vascular complications in diabetic patients.

## Additional files

Additional file 1: Figure S1. Effects of mannitol on HUVECs. HUVECs were treated with d-mannitol at the concentration of 24.5 mM in normoglycemic (5.5 mM glucose) conditions for 5 days (Mannitol). Total cell number of (left) and the ratio of cells positive for trypan blue (right) were analyzed. HUVECs cultured in normoglycemic conditions served as control (Control). n=5 for each group. Data represent means ± standard error of the mean. Additional file 2: Figure S2. Treatment with mannitol does not affect expression of PDGFR-α in endothelial cells. A: HUVECs were treated with or without 24.5 mM d-mannitol in normoglycemic conditions and then subjected to Western blot analysis for PDGFR-α. Relative quantification data are also shown (right). n=3 for each group. Data represent means ± standard error of the mean. B: HMVECs were treated with or without 24.5 mM d-mannitol in normoglycemic condition and then subjected to Western blot analysis for PDGFR-α. Relative quantification data are also shown (right). n=3 for each group. Data represent means ± standard error of the mean. Additional file 3: Figure S3. Effect of PKC inhibition on expression of PKC-α and PDGFR-α in endothelial cells. A: HUVECs exposed to 5.5 mM (Low) or 30 mM (High) glucose were treated or not bisindolylmaleimide I (Bis I) at the concentration of 2 or 4 μΜ for 30 minutes and then were subject to Western blot analysis for PKC-α and PDGFR-α expression. Relative expression levels of PKC-α and PDGFR-α are shown. **P*<0.05 vs Low glucose, Bis I (-) group. ##*P*<0.01 vs High glucose, Bis I (-) group. n=4 for each group. Data represent means ± standard error of the mean. B: HUVECs exposed to 5.5 mM (Low) or 30 mM (High) glucose were treated with or without Bis I at a concentration of 4 μΜ for 120 minutes and then were subject to Western blot analysis for PKC-α and PDGFR-α expression. Relative expression levels of PKC-α and PDGFR-α are shown. **P*<0.05, ***P*<0.01 vs Low glucose, Bis I (-) group. #*P*<0.05, ##*P*<0.01 vs High glucose, Bis I (-) group. n=4 for each group. Data represent means ± standard error of the mean. Additional file 4: Figure S4. Effects of PKC inhibition on intracellular signaling induced by VEGF-A in normoglycemic or hyperglycemic endothelial cells. HUVECs exposed to 5.5 mM (Low) or 30 mM (High) glucose were treated with VEGF-A alone (50 ng/mL) or VEGF-A + Bis I (4 μΜ) and analyzed for the VEGF-A signaling pathways by Western blot analysis. Relative expression levels of pERK and pAkt are shown as the ratio of pAkt to Akt densities, and pERK to ERK densities, respectively. **P*<0.05, ***P*<0.01 vs VEGF-A (-), Bis I (-) group. #*P*<0.05 vs VEGF-A (+), Bis I (-) group. n=5~6 for each group. Data represent means ± standard error of the mean.
