# Supplementary figures and images for: Inhibition of protein kinase C enhances angiogenesis induced by platelet-derived growth factor C in hyperglycemic endothelial cells
Source: Cardiovasc Diabetol. 2015 Feb 7;14:19. doi: 10.1186/s12933-015-0180-9 (PMC4334399; doi:10.1186/s12933-015-0180-9)

Supplemental figure 1

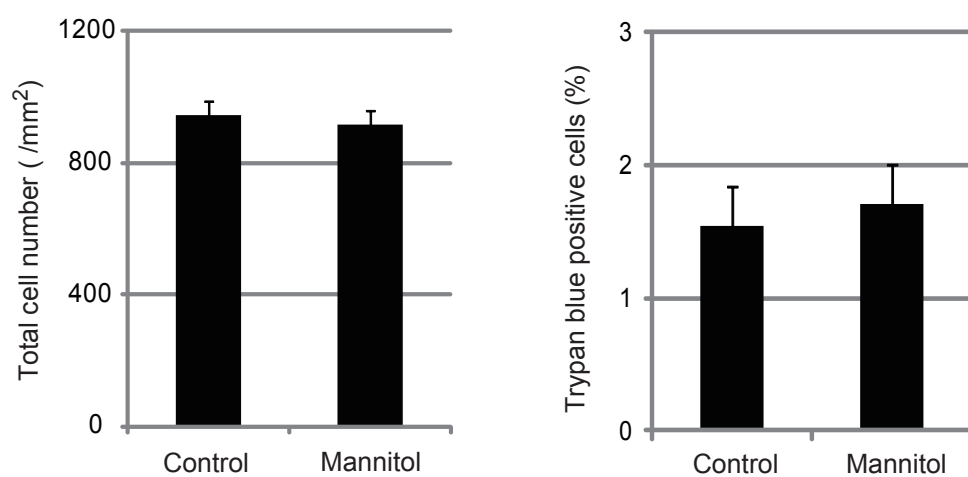

Supplement: Additional file 1: Figure S1. — Effects of mannitol on HUVECs. HUVECs were treated with d-mannitol at the concentration of 24.5 mM in normoglycemic (5.5 mM glucose) conditions for 5 days (Mannitol). Total cell number of (left) and the ratio of cells positive for trypan blue (right) were analyzed. HUVECs cultured in normoglycemic conditions served as control (Control). n=5 for each group. Data represent means ± standard error of the mean. [file 12933_2015_180_MOESM1_ESM.pdf]

Supplemental figure 2

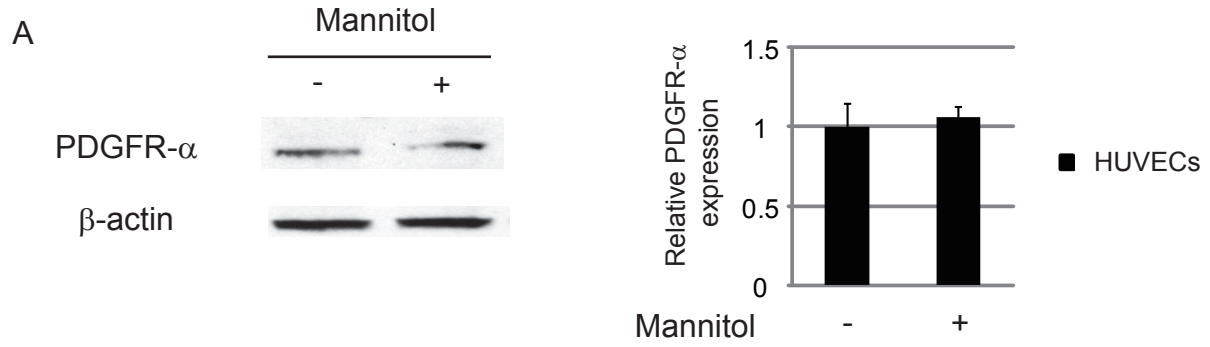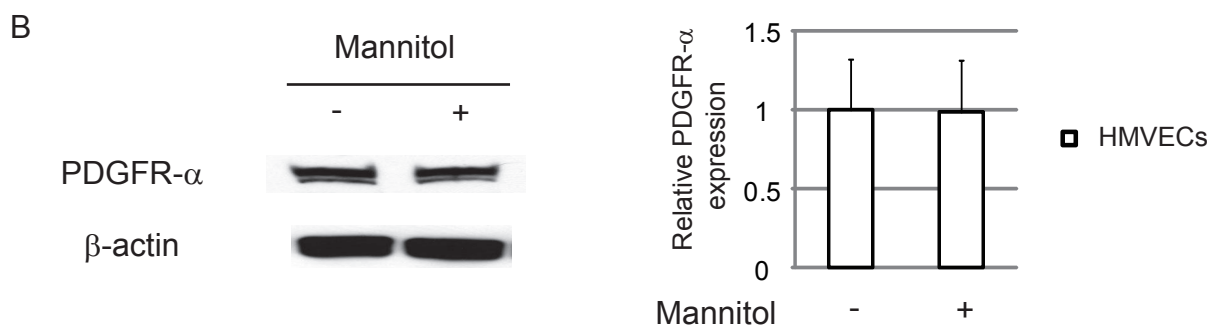

Supplement: Additional file 2: Figure S2. — Treatment with mannitol does not affect expression of PDGFR-α in endothelial cells. A: HUVECs were treated with or without 24.5 mM d-mannitol in normoglycemic conditions and then subjected to Western blot analysis for PDGFR-α. Relative quantification data are also shown (right). n=3 for each group. Data represent means ± standard error of the mean. B: HMVECs were treated with or without 24.5 mM d-mannitol in normoglycemic condition and then subjected to Western blot analysis for PDGFR-α. Relative quantification data are also shown (right). n=3 for each group. Data represent means ± standard error of the mean. [file 12933_2015_180_MOESM2_ESM.pdf]

Supplemental figure 3

A

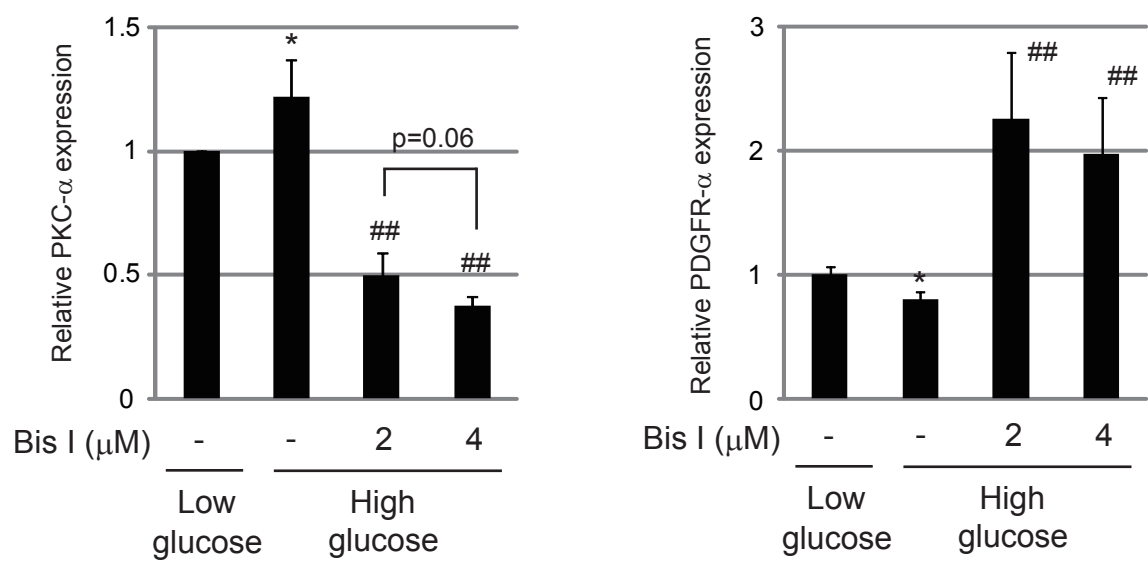

B

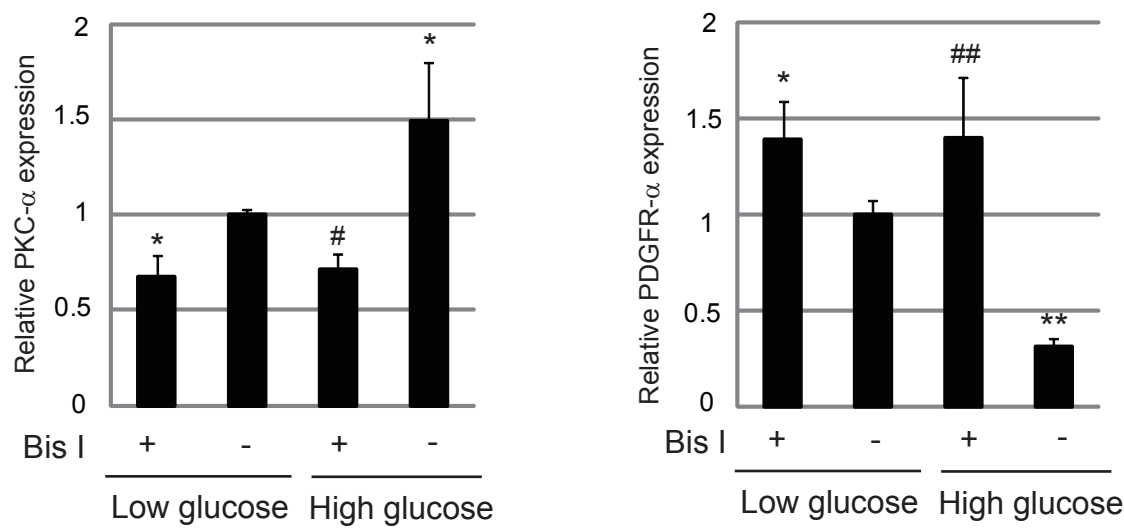

Supplement: Additional file 3: Figure S3. — Effect of PKC inhibition on expression of PKC-α and PDGFR-α in endothelial cells. A: HUVECs exposed to 5.5 mM (Low) or 30 mM (High) glucose were treated or not bisindolylmaleimide I (Bis I) at the concentration of 2 or 4 μΜ for 30 minutes and then were subject to Western blot analysis for PKC-α and PDGFR-α expression. Relative expression levels of PKC-α and PDGFR-α are shown. *P<0.05 vs Low glucose, Bis I (-) group. ##P<0.01 vs High glucose, Bis I (-) group. n=4 for each group. Data represent means ± standard error of the mean. B: HUVECs exposed to 5.5 mM (Low) or 30 mM (High) glucose were treated with or without Bis I at a concentration of 4 μΜ for 120 minutes and then were subject to Western blot analysis for PKC-α and PDGFR-α expression. Relative expression levels of PKC-α and PDGFR-α are shown. *P<0.05, **P<0.01 vs Low glucose, Bis I (-) group. #P<0.05, ##P<0.01 vs High glucose, Bis I (-) group. n=4 for each group. Data represent means ± standard error of the mean. [file 12933_2015_180_MOESM3_ESM.pdf]

Supplemental figure 4

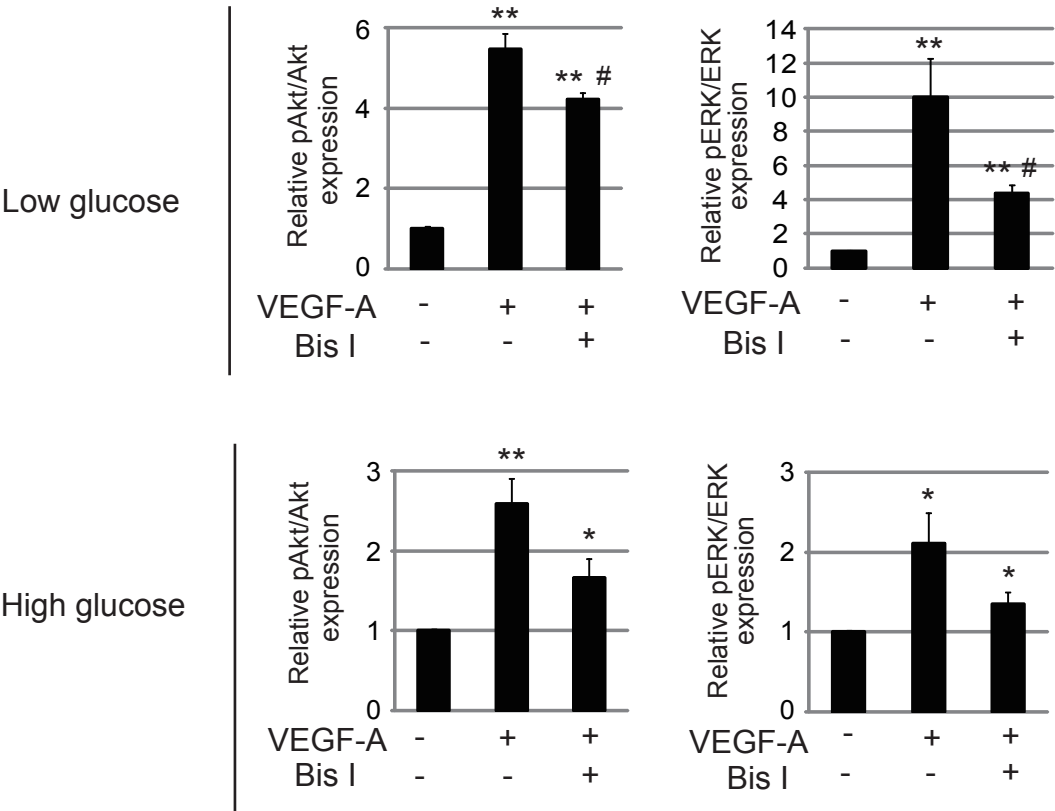

Supplement: Additional file 4: Figure S4. — Effects of PKC inhibition on intracellular signaling induced by VEGF-A in normoglycemic or hyperglycemic endothelial cells. HUVECs exposed to 5.5 mM (Low) or 30 mM (High) glucose were treated with VEGF-A alone (50 ng/mL) or VEGF-A + Bis I (4 μΜ) and analyzed for the VEGF-A signaling pathways by Western blot analysis. Relative expression levels of pERK and pAkt are shown as the ratio of pAkt to Akt densities, and pERK to ERK densities, respectively. *P<0.05, **P<0.01 vs VEGF-A (-), Bis I (-) group. #P<0.05 vs VEGF-A (+), Bis I (-) group. n=5~6 for each group. Data represent means ± standard error of the mean. [file 12933_2015_180_MOESM4_ESM.pdf]
